# Resource Utilization and Cost-Effectiveness of Counselor- vs. Provider-Based Rapid Point-of-Care HIV Screening in the Emergency Department

**DOI:** 10.1371/journal.pone.0025575

**Published:** 2011-10-12

**Authors:** Rochelle P. Walensky, Bethany L. Morris, William M. Reichmann, A. David Paltiel, Christian Arbelaez, Laurel Donnell-Fink, Jeffrey N. Katz, Elena Losina

**Affiliations:** 1 Division of Infectious Disease, Department of Medicine, Massachusetts General Hospital, Boston, Massachusetts, United States of America; 2 Division of General Medicine, Department of Medicine, Massachusetts General Hospital, Boston, Massachusetts, United States of America; 3 Division of Infectious Disease, Department of Medicine, Brigham and Women's Hospital, Boston, Massachusetts, United States of America; 4 Division of Rheumatology, Immunology and Allergy, Department of Medicine, Brigham and Women's Hospital, Boston, Massachusetts, United States of America; 5 Department of Orthopedic Surgery, Brigham and Women's Hospital, Boston, Massachusetts, United States of America; 6 Department of Emergency Medicine, Brigham and Women's Hospital, Boston, Massachusetts, United States of America; 7 Center for AIDS Research, Harvard Medical School, Boston, Massachusetts, United States of America; 8 Yale School of Medicine, New Haven, Connecticut, United States of America; 9 Department of Biostatistics, Boston University School of Public Health, Boston, Massachusetts, United States of America; Rush University, United States of America

## Abstract

**Background:**

Routine HIV screening in emergency department (ED) settings may require dedicated personnel. We evaluated the outcomes, costs and cost-effectiveness of HIV screening when offered by either a member of the ED staff or by an HIV counselor.

**Methods:**

We employed a mathematical model to extend data obtained from a randomized clinical trial of provider- vs. counselor-based HIV screening in the ED. We compared the downstream survival, costs, and cost-effectiveness of three HIV screening modalities: 1) no screening program; 2) an ED provider-based program; and 3) an HIV counselor-based program. Trial arm-specific data were used for test offer and acceptance rates (provider offer 36%, acceptance 75%; counselor offer 80%, acceptance 71%). Undiagnosed HIV prevalence (0.4%) and linkage to care rates (80%) were assumed to be equal between the screening modalities. Personnel costs were derived from trial-based resource utilization data. We examined the generalizability of results by conducting sensitivity analyses on offer and acceptance rates, undetected HIV prevalence, and costs.

**Results:**

Estimated HIV screening costs in the provider and counselor arms averaged $8.10 and $31.00 per result received. The *Provider* strategy (compared to no screening) had an incremental cost-effectiveness ratio of $58,700/quality-adjusted life year (QALY) and the *Counselor* strategy (compared to the *Provider* strategy) had an incremental cost-effectiveness ratio of $64,500/QALY. Results were sensitive to the relative offer and acceptance rates by strategy and the capacity of providers to target-screen, but were robust to changes in undiagnosed HIV prevalence and programmatic costs.

**Conclusions:**

The cost-effectiveness of provider-based HIV screening in an emergency department setting compares favorably to other US screening programs. Despite its additional cost, counselor-based screening delivers just as much return on investment as provider based-screening. Investment in dedicated HIV screening personnel is justified in situations where ED staff resources may be insufficient to provide comprehensive, sustainable screening services.

## Introduction

The revised Centers for Disease Control and Prevention (CDC) guidelines on HIV screening in 2006 recommend routine HIV testing in a variety of health care settings [1]. Though prescriptive with regard to who should be tested, the guidelines are comparatively vague with regard to who should conduct the testing. Studies of alternative models whereby HIV counselors [2], emergency department physicians [2], medical students [3] or nurses [2], [4] have each had the primary responsibility for conducting HIV testing report variable success in terms of testing offer and acceptance rates and yield of case identification. However, no studies to date have reported on the economic efficiency of these alternative approaches. Our objective was to evaluate the downstream cost-effectiveness of provider-based vs. counselor-based HIV screening, using data obtained from a recently completed clinical trial comparing these two approaches.

## Methods

### Ethics Statement

The study was approved by the Partners Human Research Committee (Protocol #2006-P-000136) and overseen by a Data Safety and Monitoring Board.

### Analytic Overview

We employed a mathematical simulation model to extend data obtained from a recently completed randomized clinical trial of provider- vs. counselor-based HIV screening [5]–[10]. The model was used to estimate the survival, downstream resource use, and incremental cost-effectiveness of the following three rapid, point-of-care ED HIV screening modalities: 1) no screening program (for comparison); 2) an ED provider-based HIV screening strategy (*Provider*); and 3) a counselor-based HIV screening strategy (*Counselor*). Data reported directly from the trial included: rates of test offer (defined as the percentage of patient encounters during which the provider explicitly offered a test); rates of test acceptance (defined as the percentage of instances where patients agreed to be tested following the offer of a test); and personnel-related direct costs [10]. Although we rely on testing rates and cost data obtained from the USHER Trial, we specifically do not use data from the USHER Trial on case detection as the trial was not powered to detect a difference in HIV detection rates by trial arm. USHER testing rates and cost data served as input parameters (Table 1) to a model-based estimation of per person quality-adjusted life expectancies and the projected per person lifetime costs. In accordance with current standards for the conduct of economic evaluation in health and medicine, all outcomes were measured from the societal perspective and discounted at 3% per year [11]. Incremental cost-effectiveness was reported in 2009 US dollars per quality-adjusted life-year gained. We conducted sensitivity analyses, varying base case input parameter values within plausible ranges to examine their influence on the overall results.

**Table 1:** Input parameters for model-based analyses.

Variable	Base Case Value	Range Examined	Reference
**Baseline cohort characteristics**			
Undiagnosed HIV prevalence (%)			[10]
Total	0.4	0.1–1.0	
Age, mean years (SD)	37 (14)	27–47	[10]
Sex			[10]
Male (%)	35		
Distribution of initial CD4, median cells/µl (IQR, SD)			
Chronic HIV infection*	467 (606, 471)		[10]
Discount Rate (annual)	3%	0–3%	
HIV RNA distribution in chronic HIV infection (%)			[15], [31]
>100,000 copies/ml	12.9		
30,001–100,000 copies/ml	12.9		
10,001–30,000 copies/ml	25.0		
3,001–10,000 copies/ml	25.2		
501–3,000 copies/ml	16.3		
<500 copies/ml	7.7		
**HIV testing protocols**			
Average background HIV test frequency	Every 5 yrs	Every 3–7 yrs	[8]
Sensitivity† (%)	99.6		[8], [32]–[35]
Specificity† (%)	97.5		[8], [32]–[35]
***Provider*** ** strategy**			
Test offer probability (%)	36	30–100	[10]
Test acceptance probability (%)‡	75	30–100	[10]
Probability of HIV-detected to link to care (%)	80	50–100	[10]
***Counselor*** ** strategy**			
Test offer probability (%)	80	30–100	[10]
Test acceptance probability (%)‡	71	30–100	[10]
Probability of HIV-detected to link to care (%)	80	50–100	[10]
**Costs (2009 US$)**			
Routine care (range by CD4, monthly) , off ART	290–2,380		[37]–[39]
Routine care (range by CD4, monthly), on ART	240–1,080		[37]–[39]
CD4 test	70		[40]
HIV RNA test	120		[40]
Acute OI events			[37]–[39]
*Pneumocystis jiroveci* pneumonia	13,120		
Mycobacterium avian complex	5,620		
Toxoplasmosis	31,320		
Cytomegalovirus	8,010		
Fungal infections	8,930		
Other opportunistic infections	6,010		
Mortality (treated and untreated patients)			[37]–[39]
Any OI event	93,990		
Chronic AIDS	59,670		

SD: Standard deviation; IQR: Inter-quartile range; OI: Opportunistic infection.

*Starting CD4 cell count, on average, for prevalent cases.

† Sensitivity and specificity refer to the characteristics of a single rapid test, not the confirmatory process; test sensitivity is assumed to be 2.5% (the false positive rate) during the acute infection window period (approximately 2 months).

‡ Probability of test acceptance is conditional upon being offered a test.

### The Cost-Effectiveness of Preventing AIDS Complications (CEPAC) Model

The CEPAC Model is a mathematical simulation of the detection, natural history and treatment of HIV disease in the US [5]–[9]. The model comprises two main functions: a screening module that captures HIV detection at the population level; and a disease module that portrays the progress of HIV infection and treatment at the patient level. Details of the CEPAC model have been previously published [5]–[9].

#### CEPAC Screening Module

The function of the screening module is to determine when and if HIV disease is detected and whether a detected case is successfully linked to care. Detection can occur via one of three mechanisms: 1) presentation with an AIDS-defining opportunistic infection; 2) “background” testing, as currently occurs at sexually transmitted disease clinics, health insurance visits, in prisons, and with increased frequency in health care settings; and 3) a dedicated HIV screening program, such as that examined in the emergency department-based trial. To present a conservative analysis with regard to the attractiveness of the dedicated HIV screening program, we assume that presentation with an AIDS-defining opportunistic infection or “background” testing function with perfect test sensitivity, specificity and linkage to care. Only upon diagnosis of HIV disease and successful linkage to care do patients become eligible for HIV-related care, opportunistic infection prophylaxis, and antiretroviral therapy according to current guidelines [12], [13].

The screening module is equipped to consider alternative assumptions regarding the occurrence, success, and cost of each component of the screening encounter. Specifically, the model tracks rates of test offer, test acceptance among those offered, as well as test confirmation and linkage to care among patients with reactive results. We assume that patients encounter background HIV testing, on average, once every 5 years. That is, everyone in the population has a monthly chance of background HIV screening equal to 1/60.

#### The CEPAC Disease Module

The disease module is a “state-transition” simulation, meaning that the natural history and clinical management of HIV infection are characterized as a series of month-to-month transitions between health “states.” These health states are defined by CD4 count (>500/µL; 351–500/µL; 201–350/µL; 101–200/µL; 51–100/µL; <50/µL), HIV RNA (viral load) level (>100,000 copies/ml, 30,001–100,000 copies/ml; 10,001–30,000 copies/ml; 3,001–10,000 copies/ml; 501–3,000 copies/ml; 0–500 copies/ml) as well as treatment and opportunistic infection history. Health states are assumed to be predictive of: further disease progression; both therapeutic and adverse responses to therapy; the development of additional co-morbidities and mortality; and the resource use associated with each of these outcomes. The model is implemented as a “Monte Carlo simulation,” meaning that a random number generator and a set of estimated probabilities are used to determine the state-to-state pathway followed by an individual, hypothetical patient.

In the CEPAC disease model, higher HIV RNA levels are associated with faster rates of CD4 decline [14] while lower CD4 cell counts are associated with an increased frequency of AIDS-related opportunistic infections [15]. Primary prophylaxis against common opportunistic infections (e.g. *Pneumocystis jiroveci*, toxoplasmosis, Mycobacterium avium complex) is provided according to current guidelines [13]. Deaths in the model are attributable to HIV-related causes, opportunistic infections, or to age-, sex- and race-adjusted background mortality rates [16]–[18].

In accordance with US-based guidelines, HIV-infected patients with detected infection in the model undergo quarterly clinical evaluation with CD4 and HIV RNA laboratories [12]. Antiretroviral therapy is initiated when CD4 counts fall below <500/µl [12]. The model specifies six sequential antiretroviral regimens with progressively decreasing efficacy, defined as percent achieving HIV RNA suppression and immunologic benefit (CD4 increase, Table 2) [19]–[25]. Treatment failure and decisions regarding regimen switches are based upon virologic rebound that is detected with an HIV RNA test.

**Table 2:** Antiretroviral therapy input parameters for model-based analyses.

Variable	Base Case Value	Monthly Cost (US$)	Reference
**Antiretroviral therapy efficacy: % HIV RNA suppression at 24 weeks, mean increase in CD4 cell count at 48 weeks**			
First line	86.0	1,430	[19], [25], [30]
	190 cells/µl		
Second line	73.3	2,050	[21], [22], [30]
	110 cells/µl		
Third line	61.3	2,040	[21], [22], [30]
	121 cells/µl		
Fourth line	64.5	2,630	[20], [30]
	102 cells/µl*		
Fifth line	40.0	4,000	[23], [24], [30]
	121 cells/µl		
Sixth line	15.0	1,740	[24], [30]
	45 cells/µl		

*At 24 weeks.

### Input Parameters

Input parameters pertaining to the clinical and economic outcomes of the HIV testing process for both the counselor- and provider-based screening strategies were obtained from the Universal Screening for HIV in the Emergency Room (USHER) Trial [10], [26]–[29]. Other HIV natural history and treatment-related data were derived from public use data sets and published estimates [14]–[17], [19]–[25], [30], [31].

### The USHER Trial: data collection and analysis

The USHER Trial was a randomized trial to examine differences in counselor- vs. provider-based HIV screening in the emergency department (ED) [10]. Details of the trial protocol and clinical outcomes have been reported elsewhere [10], [26]–[29]. In brief, after being registered, triaged, and escorted to the patient care area to be evaluated for their chief medical complaint, eligible and consenting ED patients were randomized to be offered HIV screening and complete the HIV testing process either by a dedicated HIV counselor employed by the trial or by a member of the current ED staff. Eligibility criteria, Massachusetts-specific consent processes, and participant data collection instruments have been previously described [10]. Data from this trial included population age and gender distributions, undiagnosed HIV prevalence, and clinical parameters (CD4, HIV RNA) among newly detected cases (Table 1).

#### “Coverage” for each program

We defined the “coverage” of a screening program as the product of two probabilities: the likelihood that a test was offered; and the likelihood that an offered test was accepted. In the provider arm of the USHER Trial, 36% of participants were offered a test and 75% of these participants accepted. In the counselor arm of the USHER Trial, 80% of subjects were offered a test and 71% of these accepted. Thus, baseline coverage in provider- and counselor-based programs was (0.36*0.75) 27% and (0.80*0.71) 57%, respectively. We conducted sensitivity analyses using coverage levels from 9% to 100% for both programs, as implied by the parameter ranges listed in Table 1.

Testing program success hinges on linking participants with reactive results to care. Since the USHER Trial data did not suggest significant differences between study arms, we applied the 80% overall linkage to care rate observed in the trial to both programs.

#### Prevalence of undetected HIV

Among subjects tested in the provider arm, the HIV prevalence was 7/631 (1.1%); among those tested in the counselor arm the HIV prevalence was 0/1,371 (0%) [10]. Although new HIV diagnoses differed in the two arms of the USHER Trial, we did not employ these values to estimate arm-specific levels of undetected HIV prevalence for our base case analysis. Our reason for choosing to ignore this disparity was that our goal was not to examine alternative testing strategies on different targeted populations with varying underlying HIV prevalences. Instead, we examined different strategies applied to a single population with a single undiagnosed prevalence. Our base case scenario therefore assumed a more plausible underlying prevalence of 0.4%, which we applied to both test accepters and refusers in both trial arms. It is a key assumption of our analysis that both *Provider* and *Counselor* strategies involved the offer of a test to a random sampling of this underlying population.

The observed difference in the yield of new HIV cases in the two trial arms was notable enough to prompt us to conduct extensive sensitivity analysis on the prevalence assumption: first, by altering HIV prevalence in both arms simultaneously; and second, by keeping the overall HIV prevalence constant but increasing the HIV prevalence among those offered/accepting in the provider arm to 1.0%, thereby simulating provider potential capacity to target testing to persons at elevated risk of infection, as was suggested by the USHER Trial.

#### Test performance

We applied the performance characteristics of the screening test used in the USHER Trial: the OraQuick®ADVANCE™ Rapid HIV 1/2 Antibody Test, with a reported sensitivity of 99.6% and a specificity of 97.5% [8], [32]–[35]. Participants with reactive rapid test results were asked to consent to a confirmatory HIV test (EIA and Western Blot), as well as to CD4 and viral load testing to evaluate eligibility for immediate antiretroviral therapy.

#### Resource utilization: the USHER provider arm

In the provider arm, nursing assistants offered the HIV screening test, obtained written informed consent and conducted the oral point-of-care rapid test. Non-reactive results were communicated to the patient by the house officer (resident); reactive results were communicated by the attending physician in the Emergency Department [10]. We calculated resource use in the provider arm based on the following data collected from providers in the trial: minutes spent offering the HIV test; minutes spent conducting the test; whether a test result was provided to the patient (and, if so, what the result was); and minutes spent on review of the result. Table 3 provides the total number of patient-contacts for each of these events over a one-year trial period and the annual salary for each of these provider types. Annual salaries and average hourly work weeks were obtained from the Brigham and Women's Emergency Department and are consistent with national or regional averages [36]. Because different staff members in the Emergency Department conducted different aspects of these activities, these resource utilization data (mean time per patient) were converted into dollars (2009 US$) for each activity (mean cost per patient). We then multiplied the total number of annual trial participants (N, second column, Table 3) by the mean cost per patient for each activity (sixth column); adding each of the activities resulted in a sum of $3,565. To estimate the cost per result received, we divided $3,565 by 440 (the total number receiving results). We excluded downtime in the cost calculations of the provider arm, as we assumed any downtime would be filled with other clinical duties

**Table 3:** Resource utilization and costs from the USHER Trial Provider Arm.

	N (per year)	Responsible staff member	Mean annual salary (mean weekly hours)	Mean time per patient (minutes, SD)	Mean cost per patient cost (US$)	Total cost for activity for all patients (N*per patient cost) (US$)
**PROVIDER ARM**						
HIV test offer	608	Nurse Assistant	$33,280* (40)	4.44 (3.92)	1.18	720
Conducting HIV Test	440	Nurse Assistant	$33,280* (40)	20	5.33	2,347
Reviewing results (neg)	425	House Officer	$54,336† (60)	1.61 (1.63)	0.47	199
Review results (reactive)	15	Attending Physician	$210,000‡ (50)	14.85 (19.39)	19.99	300
**Total costs for all activities**						**3,565**
**Cost per result received**						**8.10**

SD: Standard deviation.

*Obtained from Brigham and Women's Hospital, Emergency Department budgets.

† Based on average salaries post-graduate year 1–4 emergency medicine resident salaries for the 2008–2009 academic year; assumes a 60-hour resident work week.

‡ Based on median BWH attending physician salary in calendar year 2008; assumes a 50-hour attending work week. Results are consistent with AAMC northeast region, emergency medicine 2008 average, when weighted by academic rank [36].

#### Resource utilization: the USHER counselor arm

In the counselor arm of the USHER Trial, all HIV screening activities were conducted by the counselor, including test offer, test development, and reporting of non-reactive and reactive test results to participants. The results were given prior to or during the patient encounter with the ED provider to address their chief clinical complaint.

Table 4 also provides the total patient encounters for each activity, as well as the mean time per patient for each encounter as collected by the data instrument in the trial. While these provide interesting comparisons to the provider data, they were not specifically used for calculating per-result received counselor costs. Instead, we were careful in the counselor arm analyses to include counselor downtime, thereby capturing all expenses incurred by hiring new personnel. Downtime also accounted for limitations resulting from insufficient ED patients to keep counselors busy to capacity at all times. For cost-related input parameters for the cost-effectiveness analysis, we therefore divided the annual counselor salary by the number of patients per year per counselor receiving test results in the counselor arm ($32,000/1,032).

**Table 4:** Resource utilization and costs from the USHER Trial Counselor Arm.

	N (per counselor per year)	Responsible staff member	Mean annual salary (mean weekly hours)	Mean time per patient (minutes, SD)	Mean cost per patient (US$)†	
**COUNSELOR ARM**						
HIV test offer	1,498	Counselor	$32,000 (40)	3.82 (3.21)	0.98	
Conducting HIV Test	1,032	Counselor	$32,000 (40)	20	5.13	
Reviewing results (neg)	1,008	Counselor	$32,000 (40)	1.51 (1.22)	0.39	
Review results (reactive)	24	Counselor	$32,000 (40)	9.83 (8.17)	2.52	
**Cost per result received** *						**31.00**

SD: Standard deviation.

*The estimate was obtained by dividing the annual counselor salary by the number of patients per year per counselor receiving test results in the counselor arm. We have intentionally applied a conservative calculation of the cost per result received in the counselor arm, by accounting for all counselor downtime.

† Costs in this column are exclusive of downtime; this column multiplies the mean time per patient by the cost per minute of a counselor. This column is shown simply for comparison to the provider strategy and is not used in the cost-effectiveness analysis.

#### Other costs

In addition to the immediate costs of the testing program, the CEPAC model considers the HIV-associated direct medical resource use – above and beyond background medical care – resulting from the downstream outcomes of the testing program [37]–[41]. These costs include inpatient days, outpatient visits, laboratory tests, and medication costs. Indirect costs (e.g. patient time and lost wages) and direct non-medical costs are excluded.

## Results

### Resource utilization and costs derived from the USHER Trial

#### Provider Program

In the provider arm, an average of 4.44 minutes of a nursing assistant's time were spent offering HIV testing to each trial participant, translating to a cost of $1.18 per patient offered (Table 3). On average, nursing assistants spent 20 minutes conducting and developing the test for those who accepted, adding $5.33 per patient tested. Review of non-reactive results by house officers required an average of 1.61 minutes ($0.47/negative result), and review of reactive results by attending physicians averaged 14.85 minutes ($19.99/reactive result). Thus, the average cost per test received for the provider strategy, calculated as a weighted average of positive and negative results, was $8.10.

#### Counselor Program

Over a 12 month period, each counselor (annual salary = $32,000) tested and delivered valid results to an average of 1,032 participants; the cost per subject who received his/her test results was: ($32,000/1,032) = $31.00 (Table 4).

### Cost-effectiveness Analysis: Base Case

Discounted (undiscounted) quality-adjusted life expectancies for HIV-infected persons were 119.61, 125.88, and 132.72 (170.56, 181.37, and 193.21) months in the *No Screen*, *Provider* and *Counselor* strategies, respectively (Table 5). Improved survival in the two screening strategies also increased the projected discounted per person lifetime costs: *No Screen* = $1,040; *Provider* strategy = $1,160; and *Counselor* strategy = $1,310. The incremental cost-effectiveness ratio of the *Provider* strategy, compared to *No Screen* was $58,700/quality-adjusted life year (QALY). The incremental cost-effectiveness ratio of the *Counselor* strategy, compared to the *Provider* strategy, was $64,500/QALY.

**Table 5:** Base case cost-effectiveness analyses of *Counselor* vs. *Provider* strategies.

	Undiscounted HIV-infected QALE (months)	Undiscounted Population QALE (months)	Discounted HIV-infected QALE (months)	Discounted Population QALE (months)	DiscountedPer person Population lifetime costs ($)	Incremental cost-effectiveness ratio ($/QALY)*
**Base case**						
No screening program	170.56	364.15	119.61	218.38	1,040	–
*Provider* Strategy	181.37	364.19	125.88	218.40	1,160	58,700
*Counselor* Strategy	193.21	364.24	132.72	218.43	1,310	64,500
**“Target testing” in provider arm: 1% prevalence among those tested in that arm (0.18% among those not tested)**						
No screening program	170.56	364.15	119.61	218.38	1,040	–
*Counselor* Strategy	193.21	364.24	132.72	218.43	1,310	dominated†
*Provider* Strategy	197.37	364.26	135.19	218.44	1,330	55,600

QALE: Quality-adjusted life expectancy, QALY: quality-adjusted life year.

*Cost-effectiveness ratios using discounted per person lifetime costs and discounted per person QALE were calculated prior to rounding.

† “dominated” strategies are eliminated because they cost more and deliver fewer years of life saved than the comparative combination of strategies [11].

### Sensitivity Analyses

#### Undetected HIV Prevalence and Program Costs

**Figure 1:**
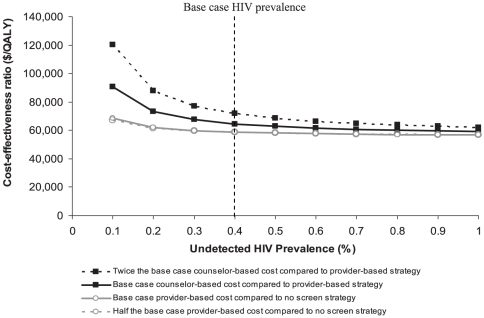
Sensitivity of incremental cost-effectiveness ratio (vertical axis) to alternative undetected HIV prevalences (horizontal axis). The incremental cost-effectiveness of the *Provider* strategy, compared to *No Screen*, is shown by the open circles. The incremental cost-effectiveness of the *Counselor* strategy, compared to the *Provider* strategy is provided by the closed squares. The dashed line (open circles) is the incremental cost-effectiveness of the *Provider* strategy, compared to *No Screen*, at half the base case provider-based screening costs ($4.05/result received). The dashed line (solid squares) is the incremental cost-effectiveness of *Counselor* strategy, compared to the *Provider* strategy, at twice the base case counselor-based screening costs ($62.00/result received).

Using program participation rates from the USHER Trial, the incremental cost-effectiveness ratio of the *Provider* strategy, compared to *No Screen*, ranged from $68,700-$56,700/QALY at HIV prevalences ranging from 0.1–1.0% (open circles, solid gray line in Figure 1). The *Counselor* strategy, compared to the *Provider* strategy, maintained an incremental cost-effectiveness ratio below $100,000/QALY at undetected HIV prevalences higher than 0.1% (solid squares, solid black line). Measured by widely accepted standards of value in health [42], this suggests a relative insensitivity to both programmatic costs and prevalence, assuming that the prevalence is balanced between arms. As undetected HIV prevalences approached 1.0%, the incremental cost-effectiveness ratio of the *Counselor* strategy approached that of the *Provider* strategy ($59,300/QALY vs. $56,700/QALY).

In a two-way sensitivity analysis on personnel costs and undetected HIV prevalence for each strategy (Figure 1, dotted lines), results were insensitive to a doubling of counselor costs ($62.00/result received, dotted line solid squares) or a halving of provider costs ($4.05/result received, dotted line, open circles).

In further sensitivity analyses, we examined the case where providers could identify persons at higher risk of HIV infection and therefore “target screen.” If the underlying prevalence among patients targeted by providers was 1% – and therefore that in the population tested by counselors was a much lower 0.18% – then the *Counselor* strategy was weakly dominated by the *Provider* strategy and the cost-effectiveness of the *Provider* strategy was slightly more attractive ($55,600/QALY, Table 5, bottom).

#### Screening Coverage by Program

**Figure 2:**
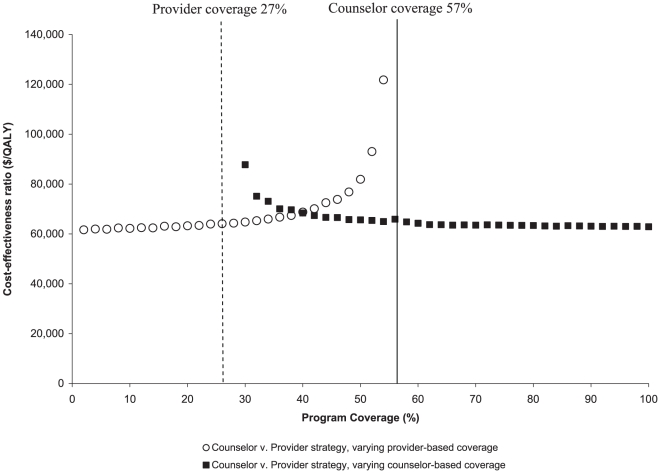
Sensitivity of incremental cost-effectiveness ratio (vertical axis) to HIV testing program coverage (horizontal axis). The squares provide the cost-effectiveness of the *Counselor* strategy compared to the *Provider* strategy at alternative rates of counselor-based program coverage; provider participation is held constant at its base case value (27%). Counselor-based testing is cost-effective at a ratio of <$100,000/QALY so long as counselor-based program coverage exceeds 30%. The circles illustrate the incremental cost-effectiveness of *Counselor* strategy to *Provider* strategy testing at alternative rates of provider-based program coverage; counselor-based coverage is held constant at its base case value (57%).

Figure 2 (solid squares) illustrates how the incremental cost-effectiveness ratio of the *Counselor* strategy varied when coverage in the *Provider* strategy was held constant at 27% and coverage in the *Counselor* strategy ranged from 28–100%. The incremental cost-effectiveness of the *Counselor* strategy sharply increased when counselor-based program coverage approached that of the *Provider* strategy and was less than 30%. When coverage in the *Counselor* strategy was instead held constant at 57% and coverage in the *Provider* strategy was varied between 2.0–54%, the incremental cost-effectiveness of the *Counselor* strategy sharply increased as coverage in the *Provider* strategy approached that in the *Counselor* strategy (i.e., >52%). When program coverage in the *Provider* strategy exceeded that in the *Counselor* strategy, the *Provider* strategy was preferred given that the *Provider* strategy was both less expensive and more effective.

#### Other Sensitivity Analyses

When the CD4 cell count of the newly identified HIV-infected cohort was lower (mean 100/µl), the incremental cost-effectiveness ratios in all strategies were more attractive (*Provider*, compared to *No Screen* $41,200/QALY; *Counselor*, compared to *Provider* $43,900/QALY). In other sensitivity analyses, the incremental cost-effectiveness results of the *Counselor* strategy were robust to the frequency of background testing (range from, on average, every 3–7 years), the average age of cohort initiation, ART efficacy, and the discount rate.

## Discussion

We used data from the USHER Trial to inform a critical, policy-relevant question regarding the revised HIV screening guidelines: does the economic efficiency of HIV screening depend on how the screening programs are designed and staffed? We found that counselor-based, compared to provider-based, routine HIV screening in an emergency department setting is cost-effective, as assessed by contemporary criteria for cost-effectiveness [11], [42]. With very similar cost-effectiveness ratios for the two rapid, point-of-care screening programs, the results also suggest roughly the same return on investment from counselor-based and provider-based screening. Why? Because, the mechanics of the screening program have little impact on either long-term costs or long-term benefits. What drives both costs and benefits in the long-run is the pathway of care and treatment triggered whenever and however a case of infection is detected and successfully linked to care. Consequently, whichever program maximizes the total number of patients linked to care is the better choice. Findings in favor of counselor-based screening were robust under assumptions intended to generalize our results beyond the USHER Trial setting including variations in undiagnosed HIV prevalence and programmatic costs.

Our results are consistent with previous studies linking the cost-effectiveness of HIV testing to the costs and benefits of downstream HIV care [7], [43]. Those studies are founded upon the grounds that HIV-infected individuals have an enormous amount of health benefit to gain from routine access to HIV care [9] – benefits that could not be realized without an HIV diagnosis. Cost-effectiveness studies also demonstrate the relative insensitivity of those findings to HIV screening costs [44]; the fact that screening costs can increase 10-fold without impacting cost-effectiveness results is due to the comparatively small component of screening costs in the overall costs of care that HIV-infected patients ultimately generate. More recent work has reported that, on a national level, the revised HIV screening guidelines will cost $2.7 billion over the next 5 years; only 18% of that budget increase is due to testing costs [45].

Although testing costs have little influence on the cost-effectiveness ratios, such costs are critical to the budgetary planning of screening program development. Indeed, a cost-effective program must also be affordable for it to be effectively implemented, and the affordability of new counseling personnel must be seriously considered. Our findings regarding the resource utilization required of emergency medicine personnel to conduct HIV testing were consistent with a survey of HIV screening costs in 45 hospitals [46]. We note that the approximate 5 minutes required for “test offer” is likely an overestimate for most other settings now that laws requiring time-intensive, written informed consent for HIV screening have been amended in most states. Among the biggest time and cost commitments in the provider arm of the USHER Trial was the delivery of reactive results by the attending physician. Though these events are relatively rare in any domestic screening program, it should be recognized that newly diagnosed HIV-infected patients will require due time and attention. This time may be hard for a busy emergency department staff to allocate but is essential to a clinically sensitive screening environment.

These results hinge upon the practical truth that in most ED settings, current staff are stretched too thin to perform point-of-care rapid HIV tests on all ED patients and to provide the spectrum of support services that such testing would require. Hiring of relatively inexpensive personnel, like HIV counselors, to improve screening coverage is worth the investment. However, if the current ED personnel have the capacity to increase coverage – such that screening program participation approaches that of the counselors – the value of additional personnel is diminished.

Results are also sensitive to the capacity of ED providers to “target” screen their patients; that is, the providers' ability to identify and test those patients at highest risk for HIV infection. While providers may excel at recognizing and testing patients who present with symptoms consistent with an AIDS-defining illness, such clinical presentations are relatively rare. Previous reports have demonstrated that providers often do not routinely nor comfortably inquire about sexual and substance use habits [47]–[50]. Some findings from the USHER Trial suggest that providers preferentially tested younger patients: the rate of test offer was similar across all ages in the counselor arm, but decreased with increasing age in the provider arm. However, among the factors measured, the trial data did not suggest differences in provider test offer rates, stratified by subjects' self-reported high sexual or substance abuse risk; differences in provider offer rates may well exist among unmeasured factors [10].

Placing program efficacy and efficiency outcomes side-by-side highlights important trade-offs demonstrated in the USHER Trial. Provider-based screening is cheaper on a per result basis. However, weaving HIV screening activities into the demands on the time of an already overstretched staff has its downsides – fewer patients may be tested. In contrast, hiring dedicated counselors for these activities ensures that a greater number of patients actually receive an HIV test but does so at greater costs. Our cost-effectiveness results suggest that there is good value to be obtained by investing in larger, counselor-based HIV case identification.

Notably, the efficacy-versus-efficiency tradeoff may become less stark in the future. With streamlined consent processes and advancing technology for non-rapid HIV tests, it may soon be feasible to HIV screen all phlebotomized emergency department patients [51], [52] without ancillary staff [53]. That such programs are still hindered by lower rates of linkage to care speaks to the continued need for counselor support and the applicability of our results.

Our results have several noteworthy limitations. First, Massachusetts remains one of three states where laws requiring written informed consent for HIV testing persist. Per person testing costs in both trial arms may be higher than those observed in states with more streamlined testing. However, all of our results point to the robustness of our findings in the face of varying program costs.

Second, we employed data obtained directly from the USHER Trial to develop base case estimates of test offer, acceptance and resource utilization parameters for both the *Provider* and *Counselor* strategies. Importantly, a single base case value of 0.4% was estimated for the prevalence of undetected HIV, and this value was applied to both the *Provider* and *Counselor* strategies in our analysis. In the base case, we chose not to make use of the difference in new HIV diagnoses observed in the two arms of the USHER Trial (7/631 in the provider arm versus 0/1371 in the counselor arm). We made this choice because we intended to examine the cost-effectiveness of alternative HIV screening strategies when applied to a single population of ED clients, not to two differing populations of such clients. Since the USHER Trial findings suggested some degree of targeting in the provider arm, we did conduct extensive sensitivity analyses assuming different prevalence values for patients served by providers and patients served by counselors. We also examined other parameters describing areas where programs may function differently, including rates of test offer and acceptance. Because the cost-effectiveness results were sensitive to the difference in prevalence by testing strategy, we urge readers to interpret our conclusions regarding the comparative value of the *Counselor* strategy – and the important impact of the potential for providers to target test – with caution.

Finally, our analysis does not capture the large potential impact of improved case detection on secondary HIV transmission. Our failure to estimate ART's preventive benefits to the broader population remains a handicap that almost certainly understates both the health benefits and cost-effectiveness findings reported here. We believe that our modeling approach can be justified on the grounds that it is conservative – i.e., taking the preventive benefits of ART into consideration would only serve to strengthen the already-favorable findings. However, we also acknowledge that there is an important qualitative difference between an attractive incremental cost-effectiveness ratio of ∼$60k/QALY and an even-more-attractive ratio several tens of thousands of dollars smaller and that this difference may, in fact, result in policy differences in some settings. Therefore, incorporating population-level transmission benefits into future analyses represents an important next step.

We found that HIV screening in the emergency department setting – whether conducted by emergency department staff or dedicated counselors – resulted in screening costs ranging from $8–$31 per test result received. While provider-based screening was cheaper on a per result-received basis, counselor-based testing ultimately screened more patients and conferred sufficient value ($64,500/QALY) to justify the additional outlay. However, cost-effectiveness does not imply affordability. In settings where resources may be insufficient to support the implementation of full-scale, counselor-based screening, provider-based approaches will continue to represent an excellent, cost-effective alternative to no screening at all.
